# Factors Affecting Mandibular Movement During Mastication in Nursing Home Residents: A Two-Year Follow-Up Study

**DOI:** 10.3390/nu18132060

**Published:** 2026-06-24

**Authors:** Enri Nakayama, Haruka Tohara, Masanori Kimura, Shinya Ohno, Fuka Shima, Iki Koide, Kimiko Abe, Kazumichi Yonenaga

**Affiliations:** 1 Department of Dysphagia Rehabilitation, Nihon University School of Dentistry, Tokyo 101-8310, Japan; 2 Department of Dysphagia Rehabilitation, Graduate School of Medical and Dental Sciences, Institute of Science Tokyo, Tokyo 1138510, Japan

**Keywords:** mandibular movement, masticatory function, nursing home residents, older adults, skeletal muscle mass, cognitive function

## Abstract

**Background/Objectives**: Declining masticatory function affects dietary variety, nutritional status, cognitive function, and health. Although factors related to chewing ability have been reported, the causes of temporal changes in masticatory kinematics in older adults remain unclear because prospective longitudinal data remain limited. Objectives: This follow-up study investigated factors associated with changes in masticatory movement in older adults requiring long-term care. **Methods**: Participants were 42 older adults residing in long-term care facilities. Survey items included mandibular kinematic data during rice cracker chewing and variables related to chewing, and the same assessment was performed two years after baseline. Relationships between changes in masticatory movement and other variables were examined, and factors associated with masticatory movement were identified using a linear mixed model (LMM). **Results**: A change in the number of cycles was significantly associated with the rate of change in the appendicular skeletal muscle mass index (ASMI). The rates of change in the number of linear motions and circular motion frequency were significantly associated with changes in the ABC Dementia Scale (ABC-DS). In the LMM results, cycle frequency remained associated with ASMI after adjustment for confounding factors, and both the number of circular motions and circular motion frequency were associated with ABC-DS. **Conclusions**: The findings suggest that masticatory movement in older adults requiring long-term care is influenced by skeletal muscle mass and cognitive function. In care facilities, interventions to maintain these factors are essential to help prevent dietary texture modifications among residents, while supporting nutrition, oral function, and health in this population.

## 1. Introduction

Numerous studies have reported on the impact of masticatory function on systemic health. Research investigating the association between masticatory ability and cognitive function in older adults has shown that a decline in chewing capacity is strongly associated with lower Mini-Mental State Examination scores. Furthermore, older adults with moderate or low masticatory ability face a significantly higher risk of developing cognitive impairment than those with high masticatory ability [1]. A decline in masticatory function associated with tooth loss tends to cause nutritional imbalances; reduce the intake of vitamins, calcium, essential fatty acids, and proteins; and may increase the risk of malnutrition due to decreased appetite [2,3]. A follow-up study of individuals aged 75 years and older reported that those with poor masticatory status have an approximately 2.5-fold higher risk of requiring long-term care after three years. These findings suggest that decreased masticatory ability is strongly associated with progression toward requiring care [4]. Moreover, regions with a higher proportion of individuals experiencing chewing difficulties have significantly lower average life expectancies for both men and women, with masticatory difficulty identified as an independent factor negatively affecting male life expectancy [5]. Consequently, maintaining masticatory ability is crucial for older adults to preserve cognitive function, nutritional status, and overall health. Older adults requiring long-term care are particularly susceptible to declining masticatory function, as factors prevalent in this population—such as dementia [6], sarcopenia [7,8], and loss of occlusal support [9]—contribute to this deterioration. Although the relationships between cognitive function, sarcopenia, occlusal support, and masticatory movement remain unclear, these factors were previously investigated by analyzing mandibular movement during rice cracker mastication in nursing home residents [10]. Analysis of mandibular masticatory cycle patterns, classified into “circular motion” and “linear motion,” revealed that cognitive decline is associated with a decrease in circular motion and an increase in linear motion. Furthermore, multivariate analysis showed a significant relationship between the proportion of circular motion and cognitive function, even after adjusting for the effects of skeletal muscle mass, occlusal support, and nutritional status. In a separate study, we reported that individuals consuming texture-modified diets showed a significantly lower proportion of circular motion during the mastication of rice crackers than those consuming regular diets [11]. These findings suggest that a reduction in the proportion of circular motion during chewing may make it difficult for care-dependent older adults with cognitive decline to maintain a normal diet. However, previous cross-sectional studies could not differentiate between preexisting masticatory habits and pathological changes. Moreover, the direction of causality between cognitive decline and altered masticatory cycles remains unclear. The primary objective of this study is to investigate the longitudinal changes in masticatory kinematics using a two-year prospective dataset and identify the underlying determinants of these changes. Ultimately, this study aims to elucidate the factors essential for the long-term preservation of masticatory function in nursing home residents, thereby informing the development of clinical strategies to minimize the necessity for dietary texture modifications in residential care facilities.

## 2. Materials and Methods

### 2.1. Participants

The initial survey was conducted between 2020 and 2021 at two long-term care facilities in Japan. Both facilities had dentists check residents’ eating habits once a month, during which facility staff could receive advice on dietary forms, eating postures, and other aspects using endoscopic swallowing evaluations if any issues related to eating were identified in residents. The inclusion criteria were as follows: (i) solid foods routinely consumed orally, and (ii) informed consent provided for the study. The exclusion criteria were as follows: (1) inability to comply with simple instructions due to cognitive impairment; (2) inability to eat while seated in a chair; (3) severe muscle weakness precluding tolerance of the examination; (4) absence of occlusal contact with molars (including dentures); and (5) history of oral cancer, painful dental conditions during mastication (including temporomandibular disorders), or denture issues. The selection of participants was carried out by the caregivers and nurses who were in charge of them on a daily basis. All the participants or their family members received oral and written explanations of the study and provided written informed consent. This study was approved by the institutional review board and conducted in accordance with the Declaration of Helsinki.

### 2.2. Observation of Masticatory Movements

The measurements were performed using methods established in previous studies [11]. A 5 mm diameter colored sticker was affixed to the most prominent anterior point of the mandible. A pharyngeal microphone (Inkou Mic NZ-210CjK, NANZU, Shimoda, Shizuoka, Japan) was attached approximately 2 cm lateral to the laryngeal prominence on the thyroid cartilage to record swallowing sounds generated as the bolus passed through the pharynx. The participants were seated in a wheelchair or armchair, wore dentures if needed, and first swallowed a sip of viscosity-adjusted water as usual. They were then given one-third of a baby rice cracker (Hyhine^®^ Baby Senbei, Kameda Seika Co., Ltd., Niigata, Japan) for practice, which becomes paste-like after several chews. Participants deemed to be at a low risk of aspiration or choking based on observations during practice proceeded to the main trial. After another sip of water, approximately 2 g of the rice cracker (Happy Turn^®^ Soft Senbei, Kameda Seika Co., Ltd., Niigata, Japan) was broken in half and provided [12]. Participants were instructed to chew while facing the camera without moving their face. An iPad (iPad Pro, Apple Inc., Cupertino, CA, USA) was positioned in front of the participant’s face to record mandibular movements and accompanying swallowing sounds from the pharyngeal microphone. To minimize stress, the examination was performed in the participant’s usual living area, and facility staff provided instructions and placed food in the mouth. Participants who moved their faces excessively or vocalized during chewing were re-recorded, and those showing no improvement were excluded.

The recorded video was analyzed by trained examiners using image analysis software (DIPP-Motion PRO 2D Ver1.25, Detect Co., Ltd., Tokyo, Japan). The trajectory of the mandibular sticker was displayed frame by frame, and the movement of the mandible was analyzed. Measurements included masticatory time (from the initial crushing sound of the rice cracker to the swallowing sound), number of cycles (each defined as the movement from the onset of mandibular depression to return to the starting position), and cycle frequency (number of cycles per masticatory time). For each cycle, motions were classified based on the mandibular locus and counted as follows. Circular motion was defined as opening centrally or slightly toward the chewing side, followed by closure, tracing a circular path. Simple linear motion was defined as opening and closing movements along the same trajectory. The numbers of each motion type were counted, and circular motion frequency (number of circular motions/total number of cycles) was calculated. Trained dentists classified and counted cycle patterns using slow-motion playback of recorded video showing the trajectories of the chin marker [10,11].

### 2.3. Other Measurements

Age, sex, activities of daily living (ADL), body mass index (BMI), skeletal muscle mass, nutritional status, dietary form, occlusal support area, and cognitive function were assessed. ADL were evaluated using the government-certified disability index (GCDI) [13]. The GCDI serves as an indicator of ADL by determining the level of care required based on assessments by nationally certified evaluators who confirm the participant’s physical condition and independence in daily life, supplemented by opinions from the primary physician. Skeletal muscle mass was evaluated using the appendicular skeletal muscle mass index (ASMI), measured via bioelectrical impedance analysis (Seca 525; Seca GmbH & Co. KG, Hamburg, Germany). Nutritional status was assessed by a dietitian using the Mini Nutritional Assessment-Short Form (MNA-SF) [14]. Dietary form was rated on a 3-level scale: normal, soft, or minced (finely chopped soft food with thickened sauce or mousse). Occlusal support area was evaluated by dentists using the Eichner index [15]. Cognitive function was assessed by physical therapists or nurses using the ABC Dementia Scale (ABC-DS) [16]. Total ABC-DS scores were categorized as normal (101–117), mild impairment (86–100), moderate impairment (71–85), and severe impairment (13–70). As only two participants scored 101 or higher, scores were grouped as mild (86–117), moderate (71–85), and severe (13–70). The most recent GCDI data were used in this study. BMI, MNA-SF, dietary form, and ABC-DS were measured within two weeks of the survey date by nurses, dietitians, and physical therapists employed at the facilities. The ASMI and Eichner index were measured on the survey date by two dentists.

### 2.4. Statistical Analysis

Changes between the initial and follow-up surveys were compared using paired t-tests for BMI and ASMI (interval scales were normally distributed, as confirmed by the Kolmogorov–Smirnov test) and Wilcoxon signed-rank tests for all the other variables. For data on masticatory movements, the percent change, which is defined as [(follow-up value − baseline value)/baseline value] × 100, was used to normalize for substantial inter-individual variability in baseline measurements. In contrast, for other variables, the absolute change (follow-up value − baseline value) was used, as these parameters allow for direct comparison regardless of their initial values. Associations were examined using Spearman’s rank correlation coefficient. To examine changes in masticatory movements between the baseline and follow-up measurements, as well as their associated factors, linear mixed model (LMM) analysis was performed. To account for intra-individual correlation resulting from the repeated-measures design, participant ID was included as a random effect (random intercept) in the models. As fixed effects, measurement time (baseline vs. follow-up) was entered as the primary explanatory variable. In addition, ASMI, Eichner index, and ABC-DS, which were selected based on previously reported associations with mastication, were included in the models as covariates to adjust for potential confounding factors. An unstructured covariance matrix was specified for the repeated-measures error structure. Statistical significance was set at *p* < 0.05. All analyses were performed using SPSS (IBM SPSS Statistics for Windows, Version 28.0; IBM Japan, Ltd., Tokyo, Japan).

## 3. Results

### 3.1. Comparison Between Baseline and Follow-Up

**Figure 1:**
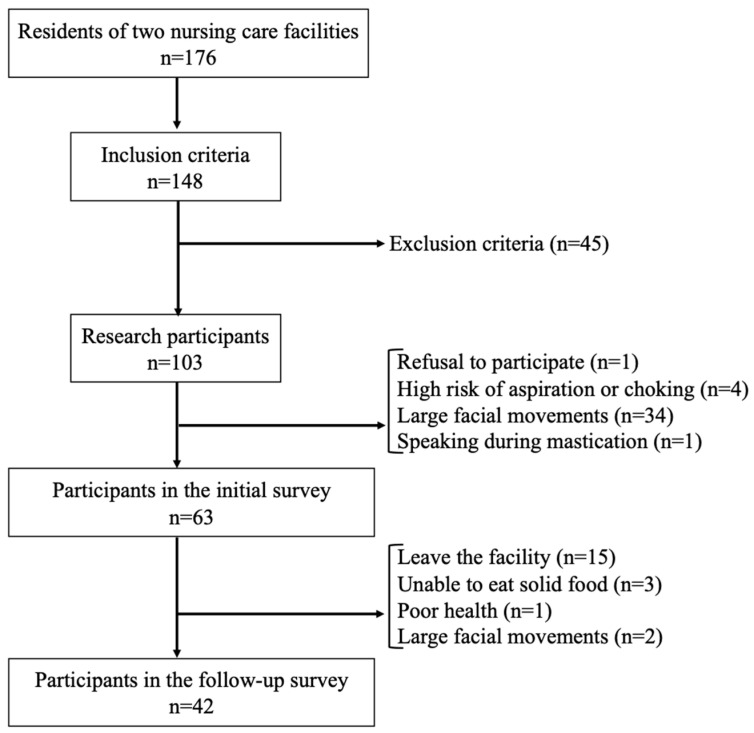
A flow chart of the participants.

Of the 176 residents in both facilities, 103 met the criteria. However, 40 were excluded from the initial survey because of poor physical condition on the measurement day or excessive facial movements during testing, resulting in the analysis of video records from 63 participants. The follow-up survey was conducted between 2022 and 2023 at the same facilities. Of the original 63 participants, 15 had been discharged because of hospitalization or death, 3 were no longer consuming solid foods, and 1 was excluded because of poor physical condition, leaving 44 eligible participants for follow-up. Of these 44 participants, two were excluded because of interruption of mastication or excessive head movement during measurement. Consequently, 42 participants (mean age 89.1 years; 11 men, 31 women) were included in the final analysis (Figure 1). Of the 42 participants, 15 had a history of cerebrovascular disease; notably, no new diseases affecting oral function developed between the initial and follow-up surveys.

Compared with baseline, follow-up results showed that 11 participants had an increase in GCDI score (indicating a decline in ADL), with a significant difference observed between the two time points (*p* < 0.003, r = 0.465). Although half of the participants showed a decrease in BMI, there was no significant difference between the two time points (*p* = 0.144, d = 0.230). Similarly, ASMI decreased in 22 participants, but there was no significant difference in the mean value (*p* = 0.808, d = 0.038). Regarding MNA-SF, the number of participants classified as malnourished significantly increased from 5 at baseline to 11 at follow-up (*p* = 0.050, r = 0.303). Eight participants showed a decrease in dietary form. In addition, the number of individuals requiring dietary form adjustments significantly increased at follow-up compared with baseline (*p* = 0.009, r = 0.400). No significant difference was found in the Eichner index, with only four participants showing a decrease (*p* = 0.066, r = 0.284). ABC-DS scores were lower in 33 participants, with a significant decrease in the median score (*p* < 0.001, r = 0.709).

Regarding masticatory parameters, a comparison of median values showed an increase in mastication time and a decrease in the number of cycles, but neither difference was statistically significant (*p* = 0.129, 0.513, r = 0.234, 0.101).

In contrast, cycle frequency decreased significantly (*p* = 0.003, r = 0.456). The number of circular motions also decreased significantly (*p* < 0.001, r = 0.572), whereas the number of linear motions increased significantly (*p* < 0.001, r = 0.587). Accordingly, the circular motion frequency also decreased significantly (*p* < 0.001, r = 0.732) (Table 1).

### 3.2. Factors Associated with Changes in Masticatory Parameters

We examined the correlations between the rates of change in masticatory parameters and changes in other parameters. There was a significant correlation between the rate of change in the number of cycles and the change in ASMI (r_s_ = −0.305, *p* = 0.049). The rate of change in the number of linear motions was significantly correlated with changes in both ABC-DS (r_s_ = −0.319, *p* = 0.040) and ASMI (r_s_ = −0.308, *p* = 0.047). In addition, the rate of change in circular motion frequency showed significant correlations with changes in ABC-DS (r_s_ = −0.329, *p* = 0.033) and the Eichner index (r_s_ = −0.318, *p* = 0.040). No significant correlations were found for the other parameters (Table 2).

### 3.3. Results of the Linear Mixed Model Analysis

To comprehensively examine changes in chewing behavior during the follow-up period, LMMs were used, with various masticatory indicators as dependent variables. In each model, the measurement time (time period) and covariates (ASMI, Eichner index, and ABC-DS) were included as fixed effects, while participants were included as a random effect (random intercept). Table 3, Table 4, Table 5 and Table 6 and Tables S1 and S2 summarize the analysis results.

The analysis revealed a significant main effect of the number of measurements on cycle frequency (F = 6.108, *p* = 0.017). The estimated fixed effects showed that follow-up measurements were significantly lower than baseline measurements (B = 0.132, *p* = 0.017). Furthermore, in the cycle frequency model, the effect of ASMI, included as a covariate, was also significant (B = 0.074, *p* = 0.027), indicating that individuals with greater skeletal muscle mass tended to have higher overall chewing frequency (Table 3). By contrast, for masticatory time and the number of cycles, the main effect of the number of measurements was not significant, and no statistically significant change was observed between baseline and follow-up measurements. None of the covariates showed a significant association in these models (Supplementary Tables S1 and S2).

Next, analysis of the masticatory cycle pattern revealed a significant main effect of the number of measurements on the number and frequency of circular motions (F = 10.153, 22.897, *p* = 0.003, <0.001). The estimated fixed effects showed that follow-up measurements were significantly lower than baseline measurements (B = 5.537 and 12.040, *p* = 0.003 and <0.001). Furthermore, the effect of ABC-DS, which was included as a covariate, was significant (B = 0.162, 0.390, *p* <0.001, <0.001), indicating that individuals with higher cognitive function tended to have a higher number of circular motions and higher circular motion frequency. For the number of linear motions, a significant main effect of the number of measurements was observed (F = 11.391, *p* = 0.001), and the estimated fixed effect showed that follow-up measurements were significantly higher than baseline measurements (B = −5.633, *p* = 0.001). However, the effect of ABC-DS, which was included as a covariate, was not significant (*p* = 0.066) (Table 4, Table 5 and Table 6).

## 4. Discussion

In this study of nursing home residents, we investigated longitudinal changes in masticatory movements during rice cracker consumption and factors associated with these changes. An increase in the number of cycles was significantly associated with the rate of change in ASMI, while changes in the number of linear motions and circular motion frequency were significantly associated with changes in ABC-DS. LMM analysis showed a significant association between cycle frequency and ASMI after adjustment for confounding factors. Additionally, the number of circular motions and circular motion frequency were associated with ABC-DS.

### 4.1. Longitudinal Changes

Comparison of baseline and follow-up surveys revealed significant declines in MNA-SF scores and dietary form. Although the mean BMI did not differ significantly, half of the participants had decreased BMI. Among participants without weight loss, 9 consumed a normal diet and 4 consumed a minced diet. Among those with weight loss, 4 consumed a normal diet and 9 consumed a minced diet; of these 9, 4 had been downgraded from a normal diet to minced food. Texture-modified diets can lead to poorer nutritional status, reduced meal satisfaction, and weight loss [17]. Although maintaining masticatory ability is crucial for preserving dietary form [18], participants showed decreased cycle frequency, reduced circular motion, and increased linear motion at follow-up. These changes may make efficient bolus formation difficult during normal diet consumption [11]. Furthermore, ABC-DS scores declined significantly. Approximately three-quarters of participants showed lower cognitive function than two years earlier, suggesting that cognitive function may decline more easily than other indicators in care facility residents.

### 4.2. Influence of Skeletal Muscle Mass on Number of Cycles and Cycle Frequency

To investigate factors associated with changes in masticatory movements, correlation analysis and LMM-based analysis were performed. An inverse correlation was found between the rate of change in the number of cycles and the change in ASMI. After adjustment for other factors, cycle frequency was associated with ASMI. ASMI, defined as appendicular skeletal muscle mass divided by height squared, is a recognized indicator for diagnosing and classifying sarcopenia severity. The Asian working group for sarcopenia (AWGS) 2019 recommends ASMI thresholds of less than 7.0 kg/m^2^ for men and 5.7 kg/m^2^ for women when using bioelectrical impedance analysis [19]. Decreased ASMI is associated with reduced physical function [20] and increased mortality risk [21], supporting its utility as an index in sarcopenia screening. Moreover, recent studies have reported associations between ASMI, masticatory ability, and tongue pressure [22]. In the follow-up survey of this study, 22 participants met the AWGS 2019 criteria, suggesting that approximately half of the participants may have had sarcopenia. Because handgrip strength, gait speed, and chair stand test results were not measured, sarcopenia could not be definitively diagnosed. These tests were omitted because many participants had cognitive decline; limiting the study to those who could complete them would have excluded individuals with moderate-to-severe dementia and prevented the study from achieving its objectives. Previous research has indicated that people with sarcopenia have weakness in the masticatory muscles (e.g., the masseter and suprahyoid muscles) and the tongue, leading to decreased occlusal force, tongue pressure, and masticatory performance [7]. Therefore, changes in participants’ masticatory parameters may have been influenced by weakness in the masticatory and tongue muscles.

A notable finding was that the number of cycles and cycle frequency showed different patterns in univariate (two-group comparison and correlation analysis) and multivariate analyses (LMM).

First, no main effect of time was observed for the number of cycles in either the two-group comparison or LMM. However, a significant negative correlation was found between chronological change in the number of cycles and change in skeletal muscle mass. These findings suggest that while the total number of cycles varies in response to intra-individual changes in skeletal muscle mass, large inter-individual variability at baseline masks this relationship, making it difficult to detect it as a uniform population-level association (fixed effect). In contrast, although cycle frequency differed significantly between the two time points, simple correlation analysis showed no association between its changes and any covariates, including skeletal muscle mass. However, when LMM was applied to control for subject-specific variability as a random effect, the main effect of time and a significant effect of skeletal muscle mass were detected. Thus, LMM may have revealed a relationship between changes in skeletal muscle mass over time and cycle frequency that was masked by individual characteristics in simple correlation analysis.

In other words, although there are differences in their characteristics, both the number of cycles and the cycle frequency were suggested to be influenced by skeletal muscle mass. An increase in the number of cycles may stimulate the satiety center and reduce food intake [23]. When bolus formation requires extensive chewing, meal time and effort increase. In older adults with sarcopenia who are prone to fatigue [24], increased meal effort may reduce intake and further exacerbate sarcopenia [25]. Thus, there is a bidirectional relationship between skeletal muscle mass and masticatory function, creating a vicious cycle that older adults are prone to falling into. Therefore, special considerations, such as modifying cooking methods, may be necessary for nursing home residents with decreased skeletal muscle mass.

### 4.3. Influence of Cognitive Function on Masticatory Cycle Patterns

Changes in the number of linear motions were inversely correlated with changes in ABC-DS; participants with more linear motions tended to have lower ABC-DS scores. Similarly, changes in circular motion frequency were positively correlated with changes in ABC-DS; participants with decreased circular motion frequency tended to have lower ABC-DS scores. Multivariate analysis using LMM showed that the number of circular motions and circular motion frequency were independently associated with cognitive function after adjustment for confounding factors. In our previous cross-sectional study, we found a relationship between cognitive function and masticatory cycle patterns, with lower cognitive function linked to lower circular motion frequency [10]. However, that study did not account for inherent individual masticatory habits, such as swallowing food with minimal chewing, which may occur regardless of cognitive status. Unlike the current longitudinal design, the cross-sectional approach did not distinguish these preexisting differences from changes associated with disease progression. This longitudinal study allowed us to account for individual baselines. Furthermore, the previous study did not determine whether changes in masticatory patterns occur as dementia progresses or precede cognitive decline. Brain dysfunction associated with dementia progression may make normal masticatory movements difficult. Conversely, if factors such as dysphagia lead to prolonged consumption of easily swallowable foods without chewing, the reduced opportunities for chewing may ultimately affect cognitive function [1,26]. Thus, the relationship between masticatory and cognitive functions is considered bidirectional. In this study, no significant correlation was found between changes in dietary form and masticatory parameters, largely ruling out the possibility that changes in dietary consistency led to a decline in chewing function and subsequently cognitive function. The relationship between changes in circular motion frequency and cognitive function persisted after adjustment for covariates. Findings suggest that the observed changes in masticatory cycle patterns are not secondary to dietary modifications, muscle atrophy, or changes in occlusal support, indicating that cognitive decline independently affects masticatory cycle patterns.

Masticatory movements are thought to be controlled by a central pattern generator in the cerebral cortex and brainstem. The facial area of the precentral cortex controls jaw and tongue movements during chewing and helps form masticatory patterns [27]. Moreover, the cortical masticatory area is essential for the smooth transition from food intake to bolus formation and swallowing [28]. In dementia, neurodegeneration and brain atrophy progress from cortical and subcortical regions and may extend to motor-related areas with disease progression. The masticatory cycle pattern changes observed in this study may reflect neurodegenerative effects on neural networks controlling mastication. However, because brain activity was not evaluated, it remains unclear whether central nervous system dysfunction related to dementia caused masticatory movement disorders.

These findings also suggest that decreased skeletal muscle mass and cognitive decline may independently affect chewing movements in residents of care facilities. Therefore, maintaining these is important for preserving efficient masticatory movements for solid food consumption. During the follow-up period, only half of the participants were able to maintain their skeletal muscle mass, and only 9 were able to maintain their cognitive function. This highlights the need for proactive interventions to maintain skeletal muscle mass and cognitive function in care facility residents.

This study had several limitations. First, because only rice crackers were used, it is unclear whether the results can be generalized. Second, although mandibular movements were measured, internal oral and pharyngeal environments were not observed; therefore, bolus formation adequacy remains unknown. Third, the sample size was smaller than planned, decreasing from 63 at baseline to 42 at follow-up. This was mainly due to COVID-19-related outbreaks in both facilities, leading to deaths and prolonged health issues that prevented residents from remaining in the care facility. Therefore, these results require cautious interpretation. Fourth, because the follow-up period was two years, longer-term follow-up may reveal other factors influencing chewing movements that were not identified in this study. Finally, although this study showed an association suggesting that masticatory patterns change with cognitive decline, these changes cannot be definitively attributed to dementia-related brain dysfunction. Further research with brain function assessments, such as fNIRS or fMRI, is necessary to verify the direct link between dementia and masticatory movement changes.

## 5. Conclusions

The number of cycles and cycle frequency appeared to be influenced by skeletal muscle mass, whereas masticatory cycle patterns appeared to be influenced by cognitive function. These findings indicate that maintaining skeletal muscle mass and cognitive function is important for preserving masticatory kinematics in long-term care residents. A decline in masticatory ability is also associated with the need for dietary texture modifications, which can affect nutritional status, cognitive function, and systemic health. Therefore, supporting skeletal muscle mass and cognitive function in care facilities is essential for maintaining oral function and promoting residents’ overall health.

## Figures and Tables

**Table 1:** Comparison of measurement items between baseline and follow-up.

	Baseline	Follow-Up	*p* Value	ES
GCDI (1/2/3/4/5)	9/7/13/9/4	4/5/10/15/8	**0.003**	0.465
BMI (kg/m^2^)	21.7 (2.6)	21.3 (2.6)	0.144	0.230
ASMI (kg/m^2^)	6.1 (1.1)	6.2 (1.2)	0.808	0.038
MNA-SF (normal/at risk/ malnutrition)	7/30/5	4/27/11	**0.050**	0.303
Dietary form (normal/soft/minced)	21/14/7	14/15/13	0.009	0.400
Eichner index (A1/A2/A3/B1/B2/B3)	32/0/1/3/5/1	30/0/2/3/4/3	0.066	0.284
ABC-DS (total score) Dementia (mild/moderate/severe)	74.5 [61.0–87.0] 12/12/18	67 [51.3–79.0] 6/13/23	**<0.001**	0.709
Kinematic measurements				
Masticatory Time (s)	26.6 [23.1–32.5]	30.3 [25.4–36.1]	0.129	0.234
Number of Cycles (n)	34.0 [29.0–42.0]	31.5 [26.0–42.0]	0.513	0.101
Cycle Frequency (n/s)	1.3 [1.1–1.6]	1.2 [0.9–1.4]	**0.003**	0.456
Number of Circular Motions (n)	23.5 [19.3–28.0]	16.5 [13.0–22.0]	**<0.001**	0.572
Number of Linear Motions (n)	12.0 [7–16]	16.0 [10.3–24]	**<0.001**	0.587
Circular Motion Frequency (%)	67.0 [60.6–77.8]	53.0 [38.5–63.4]	**<0.001**	0.732

BMI and ASMI are presented as the mean (standard deviation); other values are presented as the median [interquartile range] or numerical values. ES: Effect size, GCDI: Government-Certified Disability Index, BMI: body mass index, MNA-SF: short-form mini-nutritional assessment, ASMI: appendicular skeletal muscle index, ABC-DS: ABC dementia scale. Wilcoxon signed-rank sum test, Bold: *p* < 0.05.

**Table 2:** Correlation between the rate of change in masticatory parameters and the amount of change in other parameters.

		GCDI	BMI	MNA-SF	Dietary Form	ASMI	ABC-DS	Eichner Index
Mastication Time	r_s_	−0.072	−0.051	−0.020	0.082	−0.267	−0.234	0.020
	*p*	0.649	0.749	0.899	0.608	0.087	0.136	0.902
Number of Cycles	r_s_	0.003	−0.030	0.019	−0.047	**−0.305**	−0.104	−0.080
	*p*	0.985	0.851	0.906	0.766	0.049	0.514	0.614
Cycle Frequency	r_s_	0.078	−0.009	0.004	−0.002	−0.073	0.030	−0.160
	*p*	0.625	0.954	0.981	0.990	0.648	0.849	0.312
Number of Circular Motions	r_s_	−0.095	0.097	0.192	0.022	−0.192	0.004	−0.232
	*p*	0.551	0.543	0.224	0.889	0.223	0.978	0.139
Number of Linear Motions	r_s_	0.009	−0.077	−0.142	−0.132	**−0.319**	**−0.308**	0.090
	*p*	0.956	0.628	0.369	0.405	0.040	0.047	0.569
Circular Motion Frequency	r_s_	−0.220	0.091	0.177	0.013	0.117	**0.329**	**−0.318**
	*p*	0.162	0.568	0.262	0.935	0.462	0.033	0.040

Spearman’s rank correlation coefficient, Bold: *p* < 0.05. GCDI: Government-Certified Disability Index, BMI: body mass index, MNA-SF: short-form mini-nutritional assessment, ASMI: appendicular skeletal muscle index, ABC-DS: ABC dementia scale.

**Table 3:** Results of linear mixed model analysis for cycle frequency.

Fixed Effect/Covariate	Estimate (B)	*SE*	95% Confidence Interval	*t*	*p*-Value
Intercept	0.491	0.234	0.021–0.961	2.097	0.041
Time (Measurement)	0.132	0.054	0.025–0.240	2.471	**0.017**
ASMI	0.074	0.032	0.009–0.139	2.280	**0.027**
Eichner index	−0.010	0.023	−0.056–0.036	−0.437	0.664
ABC-DS	0.003	0.002	0.000–0.007	1.785	0.080
**Random Effect**	**Variance**	***SE***			
Participant ID (Intercept)	0.850	0.019			

Bold: *p* < 0.05. ASMI: appendicular skeletal muscle index, ABC-DS: ABC dementia scale.

**Table 4:** Results of linear mixed model analysis for the number of circular motions.

Fixed Effect/Covariate	Estimate (B)	*SE*	95% Confidence Interval	*t*	*p*-Value
Intercept	4.898	5.438	−6.055–15.851	0.901	0.373
Time (Measurement)	5.537	1.735	2.038–9.036	3.186	**0.003**
ASMI	0.147	0.750	−1.365–1.658	0.196	0.846
Eichner index	0.522	0.514	−0.516–1.560	1.016	0.316
ABC-DS	0.162	0.044	0.073–0.251	3.681	**<0.001**
**Random Effect**	**Variance**	***SE***			
Participant ID (Intercept)	73.750	16.536			

Bold: *p* < 0.05. ASMI: appendicular skeletal muscle index, ABC-DS: ABC dementia scale.

**Table 5:** Results of linear mixed model analysis for circular motion frequency.

Fixed Effect/Covariate	Estimate (B)	*SE*	95% Confidence Interval	*t*	*p*-Value
Intercept	22.430	11.221	−0.053–44.913	1.999	0.051
Time (Measurement)	12.040	2.516	6.985–17.096	4.785	**<0.001**
ASMI	0.334	1.546	−2.764–3.432	0.216	0.830
Eichner index	0.362	1.086	−1.824–2.548	0.333	0.740
ABC-DS	0.390	0.089	0.212–0.568	4.392	**<0.001**
**Random Effect**	**Variance**	***SE***			
Participant ID (Intercept)	151.558	34.416			

Bold: *p* < 0.05. ASMI: appendicular skeletal muscle index, ABC-DS: ABC dementia scale.

**Table 6:** Results of linear mixed model analysis for number of linear motions.

Fixed Effect/Covariate	Estimate (B)	*SE*	95% Confidence Interval	*t*	*p*-Value
Intercept	34.161	8.135	17.895–50.427	4.199	**<0.001**
Time (Measurement)	5.633	1.669	−8.989–−2.278	−3.375	**0.001**
ASMI	0.966	1.103	−3.176–1.243	−0.876	0.385
Eichner index	−0.589	0.819	−2.235–1.057	−0.720	0.475
ABC-DS	−0.115	0.061	−0.238–0.008	−1.882	0.066
**Random Effect**	**Variance**	***SE***			
Participant ID (Intercept)	84.518	19.355			

Bold: *p* < 0.05. ASMI: appendicular skeletal muscle index, ABC-DS: ABC dementia scale.

## Data Availability

The data that support the findings of this study are available on request from the corresponding author. The data are not publicly available due to privacy or ethical restrictions.

## Supplementary Materials

The following supporting information can be downloaded at: https://www.mdpi.com/article/10.3390/nu18132060/s1, Table S1: Results of linear mixed model analysis for mastication time; Table S2: Results of linear mixed model analysis for number of cycles.

## Abbreviations

The following abbreviations are used in this manuscript:

ASMI	Appendicular skeletal muscle mass index
ABC-DS	ABC Dementia scale
ADL	activities of daily living
GCDI	Government-certified disability index
MNA-SF	Mini nutritional assessment-short form
ES	Effect size
AWGS	Asian working group for sarcopenia
