# Supplementary material for: Factors Affecting Mandibular Movement During Mastication in Nursing Home Residents: A Two-Year Follow-Up Study
Source: Nutrients. 2026 Jun 24;18(13):2060. doi: 10.3390/nu18132060 (PMC13362863; doi:10.3390/nu18132060)
Supplement: Supplementary file 1 [file nutrients-18-02060-s001.zip › nutrients-4361317-supplementary.pdf]

Table S1. Results of linear mixed model analysis for mastication time

Fixed Effect / Covariate	Estimate (B)	*SE*	95% Confidence Interval	*t*	*p*-value
Intercept	47.712	10.680	26.294 - 69.129	4.468	< 0.001
Time (Measurement)	-4.759	2.865	-10.526 - 1.008	-1.661	0.104
ASMI	-2.074	1.449	-4.987 - 0.839	-1.431	0.159
Eichner index	0.877	1.009	-1.157 - 2.912	0.870	0.389
ABC-DS	-0.035	0.081	-0.198 - 0.127	-0.437	0.664
Random Effect	Variance	*SE*			
Participant ID (Intercept)	106.499	24.557			

ASMI: appendicular skeletal muscle index, ABC-DS: ABC dementia scale.

Table S2. Results of linear mixed model analysis for number of cycles.

Fixed Effect / Covariate	Estimate (B)	*SE*	95% Confidence Interval	*t*	*p*-value
Intercept	41.021	12.860	15.263 - 66.779	3.190	0.002
Time (Measurement)	0.168	2.795	-5.451 - 5.786	0.060	0.952
ASMI	-0.998	1.770	-4.544 - 2.549	-0.564	0.575
Eichner index	0.233	1.251	-2.286 - 2.751	0.186	0.853
ABC-DS	0.024	0.101	-0.179 - 0.228	0.240	0.810
Random Effect	Variance	*SE*			
Participant ID (Intercept)	188.555	43.955			

ASMI: appendicular skeletal muscle index, ABC-DS: ABC dementia scale.
